# Multi-Item Multiperiodic Inventory Control Problem with Variable Demand and Discounts: A Particle Swarm Optimization Algorithm

**DOI:** 10.1155/2014/136047

**Published:** 2014-06-30

**Authors:** Seyed Mohsen Mousavi, S. T. A. Niaki, Ardeshir Bahreininejad, Siti Nurmaya Musa

**Affiliations:** ^1^Department of Mechanical Engineering, Faculty of Engineering, University of Malaya, 50603 Kuala Lumpur, Malaysia; ^2^Department of Industrial Engineering, Sharif University of Technology, P.O. Box 11155-0414 Azadi Avenue, Tehran 1458889694, Iran

## Abstract

A multi-item multiperiod inventory control model is developed for known-deterministic variable demands under limited available budget. Assuming the order quantity is more than the shortage quantity in each period, the shortage in combination of backorder and lost sale is considered. The orders are placed in batch sizes and the decision variables are assumed integer. Moreover, all unit discounts for a number of products and incremental quantity discount for some other items are considered. While the objectives are to minimize both the total inventory cost and the required storage space, the model is formulated into a fuzzy multicriteria decision making (FMCDM) framework and is shown to be a mixed integer nonlinear programming type. In order to solve the model, a multiobjective particle swarm optimization (MOPSO) approach is applied. A set of compromise solution including optimum and near optimum ones via MOPSO has been derived for some numerical illustration, where the results are compared with those obtained using a weighting approach. To assess the efficiency of the proposed MOPSO, the model is solved using multi-objective genetic algorithm (MOGA) as well. A large number of numerical examples are generated at the end, where graphical and statistical approaches show more efficiency of MOPSO compared with MOGA.

## 1. Introduction and Literature Review

Most real-world problems in industries and commerce are studied as an optimization problem involving a single objective. The assumption that organizations always seek to maximize (or minimize) their profit (or their cost) rather than making trade-offs among multiple objectives has been censured for a long time. Generally, classical inventory models are developed under the basic assumption that the management purchases or produces a single product. However, in many real-life conditions, this assumption does not hold. Instead, many firms, enterprises, or vendors are motivated to store a number of products in their shops for more profitable business affairs. Another cause of their motivation is to attract the customers to purchase several items in one showroom or shop.

This work proposes a multiperiod inventory model for seasonal and fashion items. The multiperiodic inventory control problems have been investigated in depth in different research. Chiang [1] investigated a periodic review inventory model in which the period is partly long. The important aspect of his study was to introduce emergency orders to prevent shortages. He employed a dynamic programming approach to model the problem. Mohebbi and Posner [2] investigated an inventory system with periodic review, multiple replenishment, and multilevel delivery. Assuming a Poisson process for the demand, shortages were allowed in this research, and the lost sale policy could be employed. Lee and Kang [3] developed a model for managing inventory of a product in multiple periods. Their model was first derived for one item and then was extended for several products. Mousavi et al. [4] proposed a multiproduct multiperiod inventory control problem under time value of money and inflation where total storage space and budget were limited. They solved the problem using two metaheuristic algorithms, that is, genetic algorithm and simulated annealing. Mirzapour Al-e-hashem and Rekik [5] presented a multiproduct multiperiod inventory routing problem, where multiple constrained vehicles distributed products from multiple suppliers to a single plant to meet the given demand of each product over a finite planning horizon. Janakiraman et al. [6] analyzed the multiperiodic newsvendor problem and proposed some new results.

The quantity discount is of increasing attention due to its practical importance in purchasing and control of items. Usually, one derives the better marginal cost of purchase/production by taking advantages of the chances of cost savings through bulk purchase/production. Furthermore, in supply chain environments, quantity discounts can be considered an inventory coordination mechanism between buyers and suppliers [7]. In the literature of quantity discounts, Benton [8] considered an inventory system with quantity discount with multiple price breaks and alternative purchasing and lot-sizing policy. Abad [9, 10] proposed models for joint price and lot size determination when supplier offers either incremental (IQD) or all unit (AUD) quantity discounts. K. Maiti and M. Maiti [11] developed a model for a multi-item inventory control system of breakable items with AUD and IQD (and a combination of the two policies) and proposed genetic algorithm to solve the model. Sana and Chaudhuri [12] extended an EOQ model by relaxation of the preassumptions related to payments, allowing delay on delivery and discounts. They used a mixed integer nonlinear programming technique to model the problem. Taleizadeh et al. [13] considered a genetic algorithm to optimize multiproduct multiconstraint inventory control systems with stochastic replenishment intervals and discount. Recently, several works such as the ones in [4, 14–16] have also spotted discounts in inventory control problems. Huang and Lin [17] addressed an integrated model that scheduled multi-item replenishment with uncertain demand to determine delivery routes and truck loads. In this study, the products are purchased in different periods under AUD and IQD policies.

Metaheuristic algorithms have been suggested to solve some of the existing developed inventory problems in the literature. Some of these algorithms are tabu search [18, 19, 31], genetic algorithms (GA) [20–22, 32], simulating annealing (SA) [23, 33, 34], evolutionary algorithm [24, 35], threshold accepting [30], neural networks [36], ant colony optimization [37], fuzzy simulation [25], and harmony search [26, 38–41].

Inspired by social behavior of bird flocking or fish schooling, particle swarm optimization (PSO) is also a population-based stochastic optimization metaheuristic developed by Kennedy and Eberhart [42]. Recently, researchers have employed this effective technique to find optimal or near optimal solutions of their inventory control problems. For example, Taleizadeh et al. [43] employed PSO to solve their integer nonlinear programming model of a constraint joint single buyer-single vendor inventory problem with changeable lead time and (*r*, *Q*) policy in supply chains with stochastic demand. Chen and Dye [44] solved an inventory problem with deteriorating products and variable demands using a PSO algorithm. Further, Taleizadeh et al. [27] modeled a chance–constraint supply chain problem with uniformly distributed stochastic demand, where an Ant Colony Bee and a PSO algorithm were utilized to solve the problem.

Instead of optimizing a single objective, some researchers tried to find Pareto front solutions for their multiple objective inventory planning problems that usually consist of multiple conflicting objectives. Agrell [45] proposed a multicriteria framework for inventory control problem, in which the solution procedure was an interactive method with preferences extracted gradually in decision analysis process to determine batch size and security stock. Roy and Maiti [28] presented a multiobjective inventory model of deteriorating items with stock-dependent demand under limited imprecise storage area and total cost budget. Tsou [46] developed a multiobjective reorder point and order size system and proposed a multiobjective PSO (MOPSO) to generate Pareto front solutions. He employed TOPSIS to sort the nondominated solutions. The objectives therein were to maximize the profit and to minimize the wastage cost where the profit goal, wastage cost, and storage area were fuzzy in nature. One of the successful applications of PSO to MOOPs is the seminal work of Coello Coello and Lechuga [47]. Yaghin et al. [29] first addressed an inventory-marketing system to determine the production lot size, marketing expenditure, and selling prices in which the model was formulated as a fuzzy nonlinear multiobjective program. Then, they converted the model to a classical single-objective one by a fuzzy goal programming method where an efficient solution procedure using PSO was provided to solve the resulting nonlinear problem. In their study, MOPSO is not only a viable alternative to solve MOOPs, but also the only one, compared with the nondominated sorting genetic algorithm-II (NSGA-II) [48], the Pareto archive evolutionary strategy (PAES) [49], and the microgenetic algorithm [50] for multiobjective optimization problems [51].  Table 1 shows the literature review of the works reviewed in this work where DOE is an abbreviation of term “design of experiments.”

In this research, the contribution of the problem is considering a new biobjective multi-item multiperiodic inventory control model where some items are purchased under AUD and the other items are bought from IQD. The demands vary in different periods, the budget is limited, the orders are placed in batch sizes, and shortages in combination of backorder and lost sale are considered. The goal is to find the optimum inventory levels of the items in each period such that the total inventory cost and the total required warehouse space are minimized simultaneously. Since it is not easy for the managers to allocate the crisp values to the weights of the objectives in a decision making process, considering these weights as fuzzy numbers will be taken as an advantage.

In order to be more understanding of the problem, we try to explain the model with taking an example in the real world. We consider a company which produces some kinds of fashion clothes including trousers, t-shirt, and shirt in a certain period. The customers (wholesales) of this company with different demand rates make the orders and receive their products in the prespecific boxes, each one consisting of a known number of these clothes. Moreover, due to some unforeseen matters, such as production limitation, the companies are not responsive to all of the demands in a period and hence some customers must wait until the next period to receive their orders. Furthermore, it is assumed the company is going to extend the production part and therefore the owner has a plan to build and optimize a new storage subject to the available space.

The remainder of the paper is organized as follows. In Section 2, the problem along with its assumptions is defined. In Section 3, the defined problem of Section 2 is modeled. To do this, the parameters and the variables of the problem are first introduced. A MOPSO algorithm is presented in Section 4 to solve the model.  Section 5 contains a numerical example for a problem with 5 items and 3 periods, for which a multiobjective genetic algorithm (MOGA) is also applied as benchmark for comparisons. Finally, conclusion and recommendations for future research comes in Section 6.

## 2. Problem Definition, Assumptions, and Notations

Consider a biobjective multi-item multiperiod inventory control problem, in which an AUD policy is used for some items and an IQD policy for some other items. The inventory control problem of this research is similar to the seasonal items problem where the planning horizon starts in a period (or season) and finish in a certain period (or season). The total available budget in the planning horizon is limited and fixed. Due to existing ordering limitations or production constraints, the order quantities of all items in different periods cannot be more than their predetermined upper bounds. The demands of the products are constant and distinct, and, in case of shortage, a fraction is considered backorder and a fraction lost sale. The costs associated with the inventory control system are holding, backorder, lost sale, and purchasing costs. Moreover, due to current managerial decision adaptations on production policies (i.e., setting up a new manufacturing line, extending the warehouse, or building a new storage area), minimizing the total storage space is required as well as minimizing the total inventory costs. Therefore, the goal is to identify the inventory levels of the items in each period such that the two objective functions, total inventory costs and total storage space, are minimized.

In order to simplify the modeling, the following assumptions are set to the problem at hand.

1. The demand rate of an item is independent of the others and is constant in a period. However, it can be different in different periods.
2. At most one order can be placed in a period. This order can include or exclude an item.
3. The items are delivered in a special container. Thus, the order quantities must be a multiple of a fixed-sized batch.
4. The vendor uses an AUD policy for some items and an IQD policy for others.
5. A fraction of the shortages is considered backorder and a fraction lost sale.
6. The initial inventory level of all items is zero.
7. The budget is limited.
8. The planning horizon is finite and known. In the planning horizon, there are *N* periods of equal duration.
9. The order quantity on an item in a period is greater than or equal to its shortage quantity in the previous period (i.e., *Q*_*i*,*j*+1_ ≥ *b*_*i*,*j*_ defined below).

In order to model the problem at hand, in what comes next we first define the variables and the parameters. Then, the problem is formulated in Section 3.

For *i* = 1,2,…, *m* and *j* = 1,2,…, *N* − 1 and *k* = 1,2,…, *K* the variables and the parameters of the model are defined as follows:

  
*N*: number of replenishment cycles during the planning horizon,
 
*m*: number of items,
 
*K*: number of price break points,
 
*S*_*i*_: required storage space per unit of the *i*th product,
 
*T*_*j*_: total time elapsed up to and including the *j*th replenishment cycle,
 
*T*_*i*,*j*_′: *j*th period in which the inventory level of item *i* is zero (a decision variable),
 
*B*_*i*_: batch size of the *i*th product,
 
*V*_*i*,*j*_: number of the packets for the *i*th product order in period* j* (a decision variable),
 
*D*_*i*,*j*_: demand of the *i*th product in period* j*,
 
*Q*_*i*,*j*_: purchase quantity of item *i* in period *j* (a decision variable where *Q*_*i*,*j*_ = *B*_*i*_ *V*_*i*,*j*_),
 
*A*_*i*_: ordering cost per replenishment of product *i* (If an order is placed for one or more items in period *j*, this cost appears in that period),
 
*b*_*i*,*j*_: shortage quantity of the *i*th product in period *j* (a decision variable),
 
*X*_*i*,*j*_: the beginning positive inventory level of the *i*th product in period *j* (in *j* = 1, the beginning positive inventory level of all items is zero) (a decision variable),
 
*I*_*i*,*j*_: inventory position of the *i*th product in period* j* (it is *X*_*i*,*j*+1_ + *Q*_*i*,*j*+1_, if *I*_*i*,*j*_ ≥ 0, otherwise equals *b*_*i*,*j*_),
 
*I*_*i*_(*t*): the inventory level of the *i*th item at time* t*,
 
*H*_*i*_: unit inventory holding cost for item* i*,
 
*q*_*i*,*k*_:* k*th discount point for the *i*th product (*q*_*i*,1_ = 0),
 
*m*_*i*,*k*_: discount rate of item *i* in* k*th price break point (*m*_*i*,1_ = 0),
 
*P*_*i*_: purchasing cost per unit of the *i*th product,
 
*P*_*i*,*k*_: purchasing cost per unit of the *i*th product at the* k*th price break point,
 
*U*_*i*,*j*,*k*_: a binary variable, set 1 if item *i* is purchased at price break point *k* in period* j*, and 0 otherwise,
 
*W*_*i*,*j*_: a binary variable, set 1 if a purchase of item *i* is made in period* j*, and 0 otherwise,
 
*L*_*i*,*j*_: a binary variable, set 1 if a shortage for item *i* occurs in period* j*, and 0 otherwise,
 
*β*_*i*_: percentage of unsatisfied demands of the *i*th product, that is, back ordered,
 
*π*_*i*,*j*_: backorder cost per unit demand of the *i*th product in period* j*,
 
${\hat{\pi }}_{i,j}$: shortage cost per unit of the *i*th product in period *j*, that is, lost,
 
*Z*_1_: total inventory cost,
 
*Z*_2_: total storage space,
 
*TB*: total available budget,
 
*M*_1_: an upper bound for order quantity of the *i*th item in period* j*,
 
*M*_2_: an upper bound for order quantities of all items in each period (the truck capacity),
 TMF: objective function (the weighted combination of the total inventory cost and the total storage space),
 
*w*_1_: a weight associated with the total inventory cost (0 ≤ *w*_1_ ≤ 1),
 
*w*_2_: a weight associated with the total storage space (0 ≤ *w*_2_ ≤ 1).

## 3. Problem Formulation

**Figure 1:**
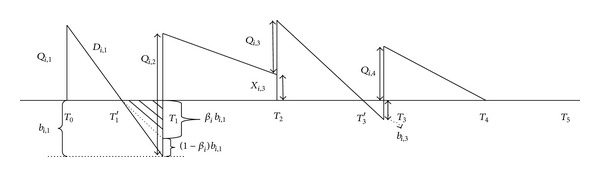
Some possible situations for the inventory of item *i* in 5 periods.

A graphical representation of the inventory control problem at hand with 5 periods for item *i* is given in Figure 1 to obtain the inventory costs. At the beginning of the primary period (*T*_0_), it is assumed the starting inventory level of item *i* is zero and that the order quantity has been received and is available. In the following periods, shortages can either occur or not. If shortage occurs, the corresponding binary variable is 1, otherwise it is zero. In the latter case, the inventory levels at the beginning of each period may be positive.

### 3.1. The Objective Functions

The first objective function of the problem, the total inventory cost, is obtained as

(1) Z1=Total  Inventory  Cost=Total  Ordering  Cost+Total  Holding  Cost +Total  Shortage  Cost+Total  Purchasing  Cost,

where each part is derived as follows.

The ordering cost of an item in a period occurs when an order is placed for it in that period. Using a binary variable *W*_*i*,*j*_, where it is 1 if an order for the *i*th product in period *j* is placed and zero otherwise, and knowing that orders can be placed in periods 1 to *N* − 1 the total ordering cost is obtained as

(2) $$\begin{array}{c} \text{Total}\;\;\text{Ordering}\;\;\text{Cost}=\overset{m}{\underset{i=1}{\sum }}\;\overset{N-1}{\underset{j=1}{\sum }}A_{i}W_{i,j}. \end{array}$$

Since it is assumed a shortage may occur for a product in a period or not, the holding cost derivation is not as straightforward as the ordering cost derivation. Taking advantage of a binary variable *L*_*i*,*j*_, where it is 1 if a shortage for item *i* in period *j* occurs and otherwise zero, and using Figure 1, the holding cost for item *i* in the time interval *T*_*j*−1_ ≤ *t* ≤ *T*_*j*_(1 − *L*_*i*,*j*_) + *T*_*i*,*j*_′*L*_*i*,*j*_ is obtained as

(3) $$\begin{array}{c} H_{i}\overset{T_{j}(1-L_{i,j})+T_{i,j}^{\prime }L_{i,j}}{\underset{T_{j-1}}{\int }}I_{i}\left(t\right)dt, \end{array}$$

where *I*_*i*_(*t*) is the inventory level of the *i*th item at time* t*.

In (3), if a shortage for item *i* occurs, *L*_*i*,*j*_ becomes 1 and the term *T*_*j*_(1 − *L*_*i*,*j*_) + *T*_*i*,*j*_′*L*_*i*,*j*_  becomes *T*_*i*,*j*_′. Otherwise, *L*_*i*,*j*_ = 0 and *T*_*j*_(1 − *L*_*i*,*j*_) + *T*_*i*,*j*_′*L*_*i*,*j*_ = *T*_*j*_. In Figure 1, the trapezoidal area above the horizontal timeline in each period when multiplied by the unit inventory holding cost of an item, *H*_*i*_, represents the holding cost of the item in that period. In other word, since

(4) $$\begin{array}{c} I_{i,j+1}=I_{i,j}+Q_{i,j}-D_{i,j} \end{array}$$

and if *I*_*i*,*j*+1_ ≥ 0 then *I*_*i*,*j*+1_ = *X*_*i*,*j*+1_, otherwise *I*_*i*,*j*+1_ = *b*_*i*,*j*_, (3) becomes

(5) Hi∫Tj−1Tj(1−Li,j)+Ti,j′Li,jIi(t)dt =Xi,j+Qi,j−Di,j2(Tj(1−Li,j)+Ti,j′Li,j−Tj−1)Hi.

Therefore, the total holding cost is obtained in

(6) Total  Holding  Cost =∑i=1m∑j=1N−1(Xi,j+Qi,j+Xi,j+12)     ×(Tj(1−Li,j)+Ti,j′Li,j−Tj−1)Hi.

The total shortage cost consists of two parts: the total backorder cost and the total lost sale cost. In Figure 1, the trapezoidal area underneath the horizontal timeline in each period (shown for the primary period) when multiplied by the backorder cost per unit demand of the *i*th product in period *j*, *π*_*i*,*j*_, is equal to the backorder cost of the item in that period. Therefore, the total backorder cost will be

(7) Total  Backorder  Cost =∑i=1m ∑j=1N−1(πi,jbi,j2(Tj−Ti,j′)βi).

Furthermore, since (1 − *β*_*i*_) represents the percentage demands of the *i*th product, that is, lost sale, the total lost sale becomes

(8) Total  Lost  Sale  Cost =∑i=1m∑j=1N−1(π^i,jbi,j2(Tj−Ti,j′)(1−βi))

in which *T*_*j*_ − *T*_*i*,*j*_′ = *b*_*i*,*j*_/*D*_*i*,*j*_.

The total purchase cost also consists of two AUD and IQD costs. The purchasing offered by AUD policy is modeled by

(9) $$\begin{array}{c} P_{i}=\{\begin{array}{cc} P_{i,1}; & 0<Q_{i,j}\leq q_{i,2}\\ P_{i,2}; & q_{i,2}<Q_{i,j}\leq q_{i,3}\\ \vdots & \\ P_{i,K}; & q_{i,K}<Q_{i,j}. \end{array} \end{array}$$

Hence, the purchasing cost of this policy is obtained as

(10) AUD  Purchasing  Cost  =∑i=1m∑j=1N−1∑k=1KPi,kQi,jUi,j,k.

**Figure 2:**
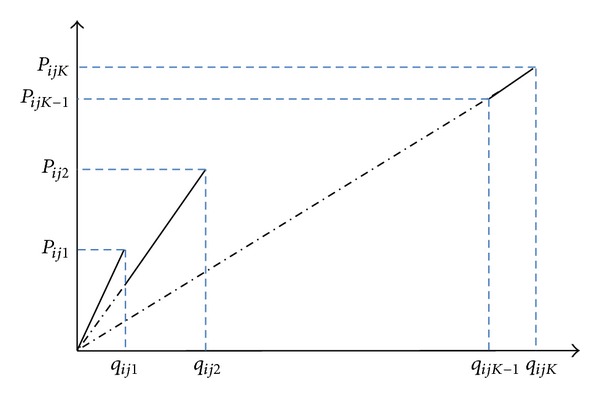
AUD policy for purchasing the products in different periods.

A graphical representation of the AUD policy employed to purchase the products in different periods is shown in Figure 2. In this Figure, the relation between the price break points and the purchasing costs is demonstrated clearly. Moreover, *U*_*i*,*j*,*k*_ is a binary variable, set 1 if the *i*th item is purchased with price break *k* in period *j* and 0 otherwise.

In the IQD policy, the purchasing cost per unit of the *i*th product depends on its order quantity. Therefore, for each price break point we have

(11) Pi,1Qi,j; 0<Qi,j≤qi,2Pi,1qi,2+Pi,2(Qi,j−qi,2)(1−mi,2); qi,2<Qi,j≤qi,3⋮Pi,1qi,2+Pi,2(qi,3−qi,2)(1−mi,2)  +⋯+Pi,K(Qi,j−qi,K)(1−mi,K);qi,K<Qi,j.

Hence, the total purchasing cost under the IQD policy is obtained as

(12) IQD  Purchasing  Cost =∑i=1m ∑j=1N−1{(Qi,j−qi,K)PiUi,j,K(1−mi,K)      +∑k=1K−1(qi,k+1−qi,k)Pi(1−mi,k)}.

**Figure 3:**
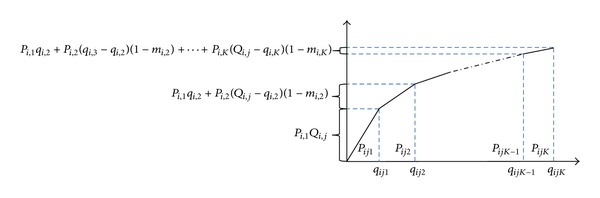
IQD policy for purchasing the products in different periods.

Figure 3 graphically depicts the IQD policy for each product in different periods.

Thus, the first objective function of the problem at hand becomes

(13) Z1=∑i=1m ∑j=1N−1AiWi,j+∑i=1m ∑j=1N−1(Xi,j+Qi,j+Xi,j+12)×(Tj(1−Li,j)+Ti,j′Li,j−Tj−1)Hi+∑i=1m ∑j=1N−1(πi,jbi,j2(Tj−Ti,j′)βi)+∑i=1m ∑j=1N−1(π^i,jbi,j2(Tj−Ti,j′)(1−βi))+∑i=1m ∑j=1N−1 ∑k=1KQi,jPi,kUi,j,k+∑i=1m ∑j=1N−1{(Qi,j−qi,K)PiUi,j,K(1−mi,K)     +∑k=1K−1(qi,k+1−qi,k)Pi(1−mi,k)}.

The second objective of the problem is to minimize the total required storage space. Since in each period, order quantities *Q*_*i*,*j*_ enter the storage and the beginning inventory of a period is the remainder inventory of the previous period, *X*_*i*,*j*_, the second objective function of the problem is modeled by

(14) $$\begin{array}{c} Z_{2}=\overset{m}{\underset{i=1}{\sum }}\;\overset{N-1}{\underset{j=1}{\sum }}\left(X_{i,j}+Q_{i,j}\right)S_{i}. \end{array}$$

Finally, the fitness function is defined as the weighted combination of the total inventory cost and the required storage space as

(15) $$\begin{array}{c} \text{TMF}=w_{1}Z_{1}+w_{2}Z_{2}. \end{array}$$

### 3.2. The Constraints

In real-world inventory planning problems, due to existing constraints on either supplying or producing goods (e.g., budget, labor, production, carrying equipment, and the like), objectives are not met simply. This section presents formulations for some real-world constraints.

The first limitation is given in (4), where it relates the beginning inventory of the items in a period to the beginning inventory of the items in the previous period plus the order quantity of the previous period minus the demand of the previous period.

The second limitation is due to delivering the items in packets of batches. Since *Q*_*i*,*j*_ represents the purchase quantity of item *i* in period *j*; denoting the batch size by *B*_*i*_ and the number of packets by *V*_*i*,*j*_, we have

(16) $$\begin{array}{c} Q_{i,j}=B_{i}V_{i,j}. \end{array}$$

Furthermore, since *Q*_*i*,*j*_ can only be purchased based on one price break point, the following constraint must hold:

(17) $$\begin{array}{c} \overset{K}{\underset{k=1}{\sum }}U_{i,j,k}=1. \end{array}$$

The prerequisite of using this strategy is that the lowest *q*_*i*,*k*_ in the AUD table must be zero (i.e., *q*_*i*,1_ = 0).

Since the total available budget is TB, the unit purchasing cost of the product is *P*_*i*_, and the order quantity is *Q*_*i*,*j*_, the budget constraint will be

(18) $$\begin{array}{c} \overset{m}{\underset{i=1}{\sum }}\;\overset{N-1}{\underset{j=1}{\sum }}Q_{i,j}P_{i}\leq \text{TB}. \end{array}$$

In real-world environments, the order quantity of an item in a period may be limited. Defining *M*_1_ an upper bound for this quantity, for *i* = 1,2,…, *m* and *j* = 1,2,…, *N* − 1 we have

(19) $$\begin{array}{c} Q_{i,j}\leq M_{1}. \end{array}$$

Moreover, due to transportation contract and the truck capacity, the number of product orders and the total order quantities in a period are limited as well. Hence, for *j* = 1,2,…, *N* − 1, we have

(20) $$\begin{array}{c} \overset{m}{\underset{i=1}{\sum }}Q_{i,j}W_{i,j}\leq M_{2}, \end{array}$$

where if an order occurs for item *i* in period *j*, *W*_*i*,*j*_ = 1, otherwise *W*_*i*,*j*_ = 0. Further, *M*_2_ is an upper bound on the total number of orders and the total order quantities in a period.

As a result, the complete mathematical model of the problem is

(21) $$\begin{array}{c} \mathrm{Min}\;\text{TMF}=w_{1}Z_{1}+w_{2}Z_{2} \end{array}$$

subject to

(22)  Z1=∑i=1m ∑j=1N−1AiWi,j+∑i=1m ∑j=1N−1(Xi,j+Qi,j+Xi,j+12)×(Tj(1−Li,j)+Ti,j′Li,j−Tj−1)Hi+∑i=1m ∑j=1N−1(πi,jbi,j2(Tj−Ti,j′)βi)+∑i=1m ∑j=1N−1(π^i,jbi,j2(Tj−Ti,j′)(1−βi))+∑i=1m ∑j=1N−1 ∑k=1KQi,jPi,kUi,j,k+∑i=1m ∑j=1N−1{(Qi,j−qi,K)PiUi,j,K(1−mi,K)     +∑k=1K−1(qi,k+1−qi,k)Pi(1−mi,k)}, Z2=∑i=1m ∑j=1N−1(Xi,j+Qi,j)Si,         Ii,j+1=Ii,j+Qi,j−Di,j;(i=1,2,…,m), (j=1,2,…,N−1),Ii,j+1={Xi,j+1;Ii,j+1≥0bi,j;Ii,j+1<0;(i=1,2,…,m), (j=1,2,…,N−1),Qi,j=BiVi,j;(i=1,2,…,m), (j=1,2,…,N−1),∑k=1KUi,j,k=1;(i=1,2,…,m), (j=1,2,…,N−1),∑i=1m ∑j=1N−1Qi,jPi≤TBQi,j≤M1;(i=1,2,…,m), (j=1,2,…,N−1)Wi,j∈{0,1}; (j=1,2,…,N−1)Ui,j,k∈{0,1};(i=1,2,…,m), (j=1,2,…,N−1),  (k=1,2,…,K)∑i=1mQi,jWi,j≤M2; (j=1,2,…,N−1)Qi,j+1≥bi,j.

In most inventory-planning models that have been developed so far, researchers have imposed some unrealistic assumptions such that the objective function of the model becomes concave and the model can easily be solved by some mathematical approaches like the Lagrangian or the derivative methods. However, since the model in (22), which is obtained based on assumptions that are more realistic, is an integer nonlinear programming mixed with binary variables, reaching an analytical solution (if any) to the problem is difficult. In addition, efficient treatment of integer nonlinear optimization is one of the most difficult problems in practical optimization [52]. As a result, in the next section a metaheuristic algorithm is proposed to solve the model in (22).

## 4. The Proposed Multiobjective Particle Swarm Optimization Algorithm

Many researchers have successfully used metaheuristic methods to solve complicated optimization problems in different fields of scientific and engineering disciplines; among them, the particle swarm optimization (PSO) algorithm is one of the most efficient methods. That is why this approach is taken in this research to solve the model in (22). The structure of the proposed MOPSO that is based on the PSO algorithm for the multiobjective inventory planning problem at hand is given as follows.

### 4.1. Generating and Initializing the Particles Positions and Velocities

PSO is initialized by a group of random particles (solutions) called generation and then searches for optima by updating generations. The initial population is constructed by randomly generated *R* particles (similar to the chromosomes of a genetic algorithm). In a *d*-dimensional search space, let ${\vec{x}}_{k}^{i}=\left\{x_{k,1}^{i},x_{k,2}^{i},\ldots ,x_{k,d}^{i}\right\}$ and ${\vec{v}}_{k}^{i}=\left\{v_{k,1}^{i},v_{k,2}^{i},\ldots ,v_{k,d}^{i}\right\}$ be, respectively, the position and the velocity of particle *i* at time *k*. Then, (23) is applied to generate initial particles, in which *x*_min⁡_ and *x*_max⁡_ are the lower and the upper bounds on the design variable values and RAND is a random number between 0 and 1. Consider

(23) $$\begin{array}{c} x_{0}^{i}=x_{\min}+\text{RAND}\left(x_{\max}-x_{\min}\right)\\ v_{0}^{i}=x_{\min}+\text{RAND}\left(x_{\max}-x_{\min}\right). \end{array}$$

An important note for the generating and initializing step of the PSO is that solutions must be feasible and must satisfy the constraints. As a result, if a solution vector does not satisfy a constraint, the related vector solution will be penalized by a big penalty on its fitness.

### 4.2. Selecting the Best Position and Velocity

For every particle, denote the best solution (fitness) that has been achieved so far as

(24) $$\begin{array}{c} \vec{p{\text{best}}_{k}^{i}}=\left\{p{\text{best}}_{k,1}^{i},p{\text{best}}_{k,2}^{i},\ldots ,p{\text{best}}_{k,d}^{i}\right\}, \end{array}$$

(25) $$\begin{array}{c} \vec{g{\text{best}}_{k}^{i}}=\left\{g{\text{best}}_{k,1}^{i},g{\text{best}}_{k,2}^{i},\ldots ,g{\text{best}}_{k,d}^{i}\right\}, \end{array}$$

where $\vec{p{\text{best}}_{k}^{i}}$ in (25) is the best position already found by particle *i* until time *k* and $\vec{g{\text{best}}_{k}^{i}}$ in (24) is the best position already found by a neighbor until time *k*.

### 4.3. Velocity and Position Update

The new velocities and positions of the particles for the next fitness evaluation are calculated using [53, 54]

(26) vk+1,di=w·vk,di+C1·r1·(pbestk,di−xk,di)+C2·r2·(gbestk,di−xk,di),xk+1,di=xk,di+vk+1,di,

where *r*_1_ and *r*_2_ are random numbers between 0 and 1, coefficients *C*_1_ and *C*_2_ are given acceleration constants towards $\vec{p\text{best}}$ and $\vec{g\text{best}}$, respectively, and *w* is the inertia weight. Introducing a linearly decreasing inertia weight into the original PSO significantly improves its performance through the parameter study of inertia weight [55, 56]. Moreover, the linear distribution of the inertia weight is expressed as follows [55]:

(27) $$\begin{array}{c} w=w_{\max}-\frac{w_{\max}-w_{\min}}{\text{iter}\_\max}\text{iteration}, \end{array}$$

where iter_max⁡ is the maximum number of iterations and iteration is the current number of iteration. Equation (27) presents how the inertia weight is updated, considering *w*_max⁡_ and *w*_min⁡_ are the initial and the final weights, respectively. The parameters *w*_max⁡_ = 0.9 and *w*_min⁡_ = 0.4 that were previously investigated by Naka et al. [55] and Shi and Eberhart [56] are used in this research as well.

### 4.4. Stopping Criterion

Achieving a predetermined solution, steady-state mean, and standard deviations of a solution in several consecutive generations, stopping the algorithm at a certain computer CPU time, or stopping when a maximum number of iterations is reached are usual stopping rules that have been used so far in different research works. In current research, the PSO algorithm stops when the maximum number of iterations is reached.

Pseudocode 1 shows the pseudocode of the proposed MOPSO algorithm. Moreover, since the problem and hence the model is new and there is no other available algorithm to compare the results, a multiobjective genetic algorithm (MOGA) is developed in this research for validation and benchmarking. MOGA was coded using roulette wheel in selection operator, population size of 40, uniform crossover with probability of 0.64, one-point random mutation with probability 0.2, and a maximum number of 500 iterations.  Pseudocode 2 shows the pseudocode of the proposed MOGA algorithm. The computer programs of the MOPSO and MOGA algorithms were developed in MATLAB software and are executed on a computer with 2.50 GHz of core 2 CPU and 3.00 GB of RAM. Furthermore, all the graphical and statistical analyses are performed in MINITAB 15.

**Pseudocode 1:**
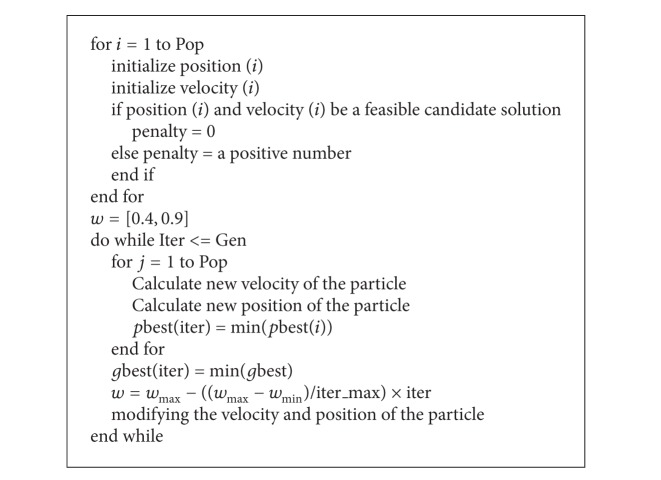
The pseudocode of MOPSO algorithm.

**Pseudocode 2:**
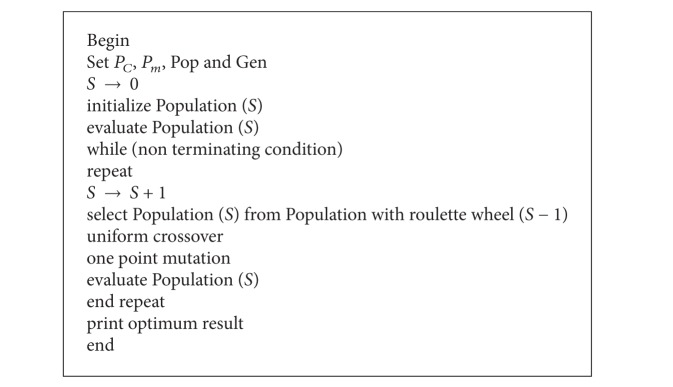
The pseudocode of MOGA algorithm.

In the next section, some numerical examples are given to illustrate the application of the proposed MOPSO algorithm in real-world environments and to evaluate and compare its performances with the ones obtained by a MOGA method.

## 5. Numerical Illustrations

The decision variables in the inventory model (22) are *Q*_*i*,*j*_, *X*_*i*,*j*_, *V*_*i*,*j*_, and *b*_*i*,*j*_. We note that the determination of the order quantity of the items in different periods, that is, *Q*_*i*,*j*_, results in the determination of the other decision variables as well. Hence, we first randomly generate *Q*_*i*,*j*_, that is, modeled by the particles' position and velocity. Equation (28) shows a pictorial representation of the matrix *Q* for a problem with 4 items in 4 periods, where rows and columns correspond to the items and the periods, respectively.

The structure of a particle

(28) $$\begin{array}{c} Q_{4,4}=\left[\begin{array}{cccc} 124 & 116 & 50 & 0\\ 205 & 190 & 58 & 0\\ 114 & 68 & 107 & 0\\ 43 & 87 & 210 & 0 \end{array}\right]. \end{array}$$

Table 2 shows partial data for 40 different problems with different sizes along with their near optimal solutions obtained by MOPSO and MOGA. In these problems, the number of items varies between 1 and 20 and the number of periods takes values between 3 and 15. In addition, the total available budgets and the upper bounds for the order quantities (*M*_1_) are given in Table 1 for each problem.

In order to illustrate how the results are obtained, consider a typical problem with 5 items and 3 periods (the seventh row in Table 2), for which the complete input data is given in Table 3. The parameters of the MOPSO and MOGA algorithms are set by Taguchi method where *C*_1_, *C*_2_ the number of populations (Pop) and number of generations (Gen) are the parameters of MOPSO and crossover probability and their level values are shown in Table 4. Furthermore, the rest of MOPSO's parameters are set as *w*_min⁡_ = 0.4, *w*_max⁡_ = 0.9 and the time periods *T*_*j*_ = 3 for *j* = 0,1, 2,3. The above parameter settings are obtained performing intensive runs. Furthermore, the amount of *V*_*i*,*j*_ will be obtained automatically after gaining the order quantity *Q*_*i*,*j*_.

**Figure 4:**
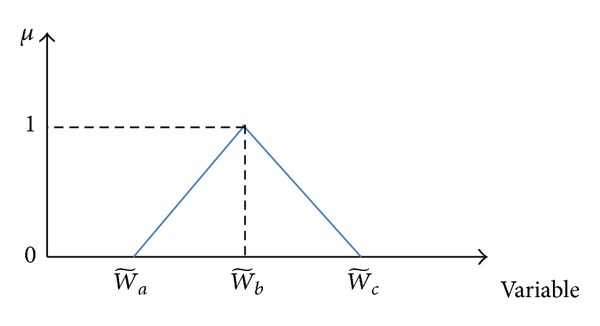
The triangular fuzzy numbers.

The weights associated with the objectives are as triangular fuzzy number $\tilde{w}=[{\tilde{w}}_{a},{\tilde{w}}_{b},{\tilde{w}}_{c}]$ shown in Figure 4 where membership function of variable *x* is given by

(29) $$\begin{array}{c} \tilde{\mu }\left(x\right)=\{\begin{array}{cc} 0 & x<{\tilde{w}}_{a}\\ \frac{x-{\tilde{w}}_{a}}{{\tilde{w}}_{b}-{\tilde{w}}_{a}} & {\tilde{w}}_{a}<x<{\tilde{w}}_{b}\\ \frac{{\tilde{w}}_{c}-x}{{\tilde{w}}_{c}-{\tilde{w}}_{b}} & {\tilde{w}}_{b}<x<{\tilde{w}}_{c}\\ 0 & {\tilde{w}}_{c}<x. \end{array} \end{array}$$

Now, in order to get crisp interval by *α*-cut operation, interval ${\tilde{w}}_{\alpha }$ can be obtained as follows (∀*α* ∈ [0,1]):

(30) $$\begin{array}{c} \frac{{\tilde{w}}_{a}^{\left(\alpha \right)}-{\tilde{w}}_{a}}{{\tilde{w}}_{b}-{\tilde{w}}_{a}}=\alpha ,\;\;\frac{{\tilde{w}}_{c}^{}-{\tilde{w}}_{c}^{\left(\alpha \right)}}{{\tilde{w}}_{c}-{\tilde{w}}_{b}}=\alpha . \end{array}$$

We have

(31) $$\begin{array}{c} {\tilde{w}}_{a}^{\left(\alpha \right)}=\left({\tilde{w}}_{b}-{\tilde{w}}_{a}\right)\alpha +{\tilde{w}}_{a};\\ {\tilde{w}}_{c}^{\left(\alpha \right)}={\tilde{w}}_{c}-\left({\tilde{w}}_{c}-{\tilde{w}}_{b}\right)\alpha . \end{array}$$

Therefore,

(32) w~α=[w~a(α),w~c(α)]=[(w~b−w~a)α+w~a,w~c(α)=w~c−(w~c−w~b)α],

where ${\tilde{w}}_{1}=\left[0.3,0.5,0.7\right]$, ${\tilde{w}}_{2}=\left[0.2,0.3,0.6\right]$, and *α* = 0.5.

**Figure 5:**
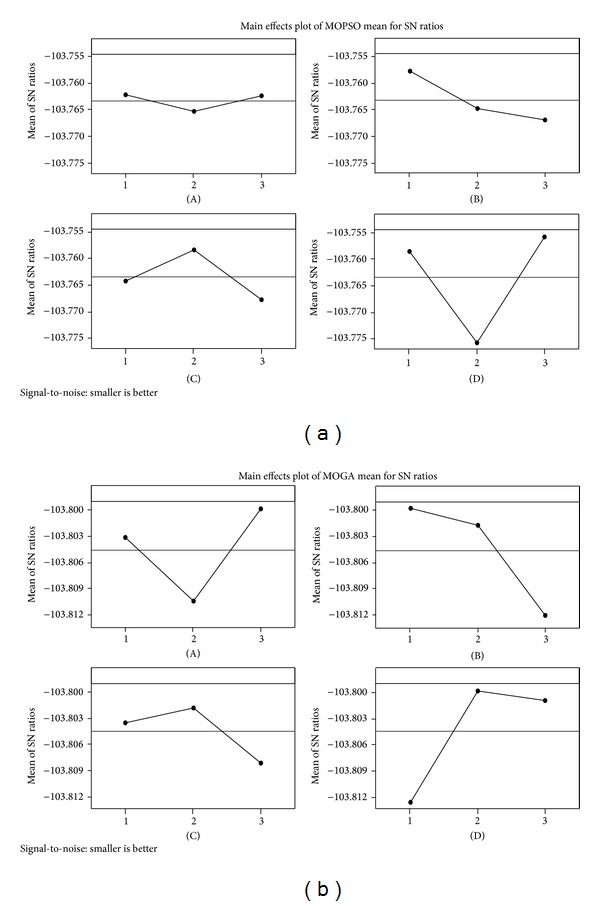
The mean S/N ratio plot for parameter levels of MOPSO and MOGA in problem 7 of Table 2.

To perform Taguchi approach in this paper, a *L*_9_ design is utilized, based on which the results for problem 7 described in Table 2 are shown in Table 5 as an example. The optimal values of the levels of the algorithms' parameters shown in Table 5 are represented by Table 6.  Figure 5 depicts the mean S/N ratio plot each level of the factors of MOPSO and MOGA for problem 7 in Table 2.

Tables 7 and 8 show the best result obtained by MOPSO and MOGA for the problem with 5 items and 3 periods (problem 7), respectively, including the amounts of decision variables and the optimal objective values. In these tables, TMF is the best value of the biobjective inventory planning problem, which is given in the last two columns of Table 2. Similarly, the best TMF for the other problems is obtained and is summarized in Table 2.

**Figure 6:**
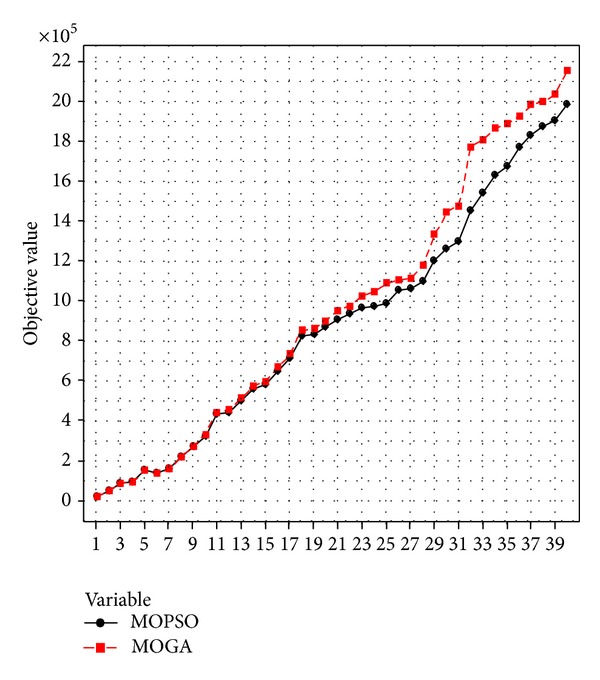
The pictorial representation of the performances of the algorithms.

To compare the performances of the MOPSO and MOGA, several statistical and graphical approaches are employed. A one-way ANOVA analysis of the means of the algorithms in confidence 0.95% is used to compare and evaluate the objective values of the generated 40 problems.  Table 9 shows the ANOVA analysis of the results of the two algorithms that demonstrates no significant difference between both algorithms. Moreover, the mean and standard deviation (Std. Dev) of the objective values of the 30 generated problems shows that the MOPSO has the better performance in terms of the objective values in comparison with the MOGA. In addition, a pictorial presentation of the performances of the two algorithms shown by Figure 6 displays that the MOPSO is more efficient than the MOGA algorithm in the large number of the problems.

**Figure 7:**
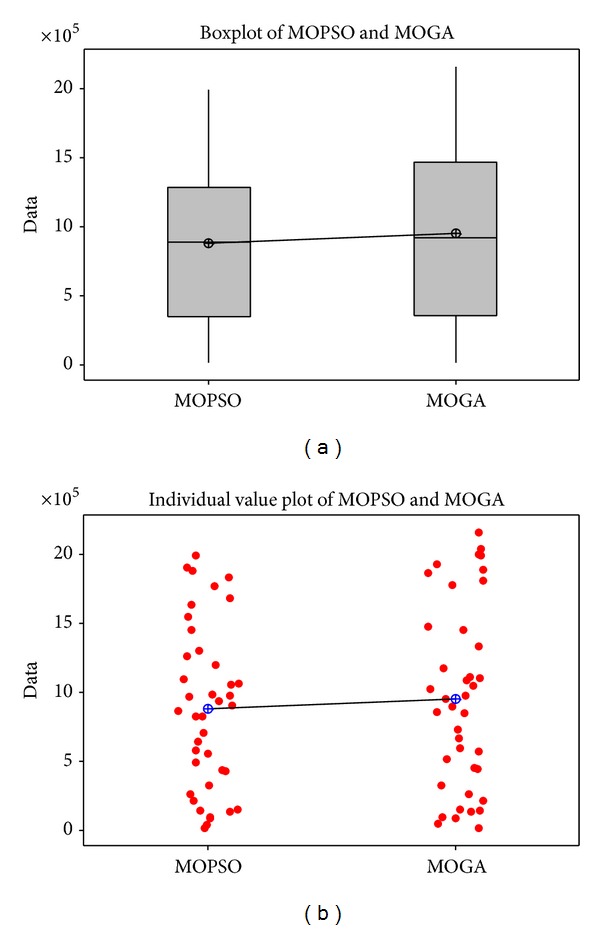
The boxplot and the individual value plot of the performances of the algorithms.

**Figure 8:**
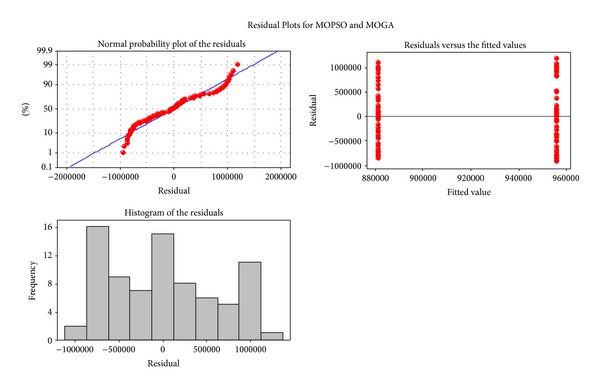
The residual plots of the algorithms.

Figure 7 depicts the boxplot and the individual value plot and Figure 8 shows the residual plots for the algorithms.

A comparison of the results in Table 2 shows that the MOPSO algorithm performs better than the MOGA in terms of the fitness functions values.

## 6. Conclusion and Recommendations for Future Research

In this paper, a biobjective multi-item multiperiod inventory planning problem with total available budget under all unit discount for some items and incremental quantity discount for other items was considered. The orders were assumed to be placed in batch sizes and the order quantities at the end period were zeros. Shortages were allowed and contain backorder and lost sale. It was assumed that the beginning inventory level in primary period was zeros and the order quantity in each period was more than the shortage quantity in the previous period. Due to adopting decisions related to a certain department of production planning (extending warehouse or building a new manufacturing line), the manager decided to build a new warehouse for the ordered items. The objectives were to minimize both the total inventory costs and the total required storage space, for which a weighted combination was defined as the objective function. The aim of the study was to determine the optimal order quantity and the shortage quantity of each product in each period such that the objective function is minimized and the constraints hold. The developed model of the problem was shown to be an integer nonlinear programming mixed with binary variables. To solve the model, both a multiobjective particle swarm optimization and a multiobjective genetic algorithm were applied. The results showed that for the 10 specific problems the MOPSO performs better than the MOGA in terms of the fitness function values.

Some recommendations for future works are to expand the model to cover a supply chain environment, to consider fuzzy or stochastic demands, and/or to take into account the inflation and the time value of the money.

## Figures and Tables

**Table 1:** Literature review of the related works.

References	Multiproduct	Multiperiod	Fuzzy multiobjective	Discount policy	Solving methodology	Shortages	DOE
[1]	—	*✓*	—	—	B and B	—	—
[2]	—	—	—	—	Level-crossing	*✓*	—
[3]	—	*✓*	—	IQD	Numerical methods	—	—
[4]	*✓*	*✓*	—	IQD	GA	—	—
[5]	*✓*	*✓*	—	—	CPLEX	—	—
[6]	—	*✓*	—	—	—	*✓*	—
[7]	—	—	—	IQD and AUD	Simulation	—	—
[8]	—	—	—	AUD	Simulation	—	—
[9]	—	—	—	IQD	Numerical methods	—	—
[10]	—	—	—	IQD	Numerical methods	—	—
[11]	*✓*	—	—	IQD and AUD	GA	—	—
[12]	*✓*	—	—	AUD	Numerical methods	—	—
[13]	*✓*	—	—	IQD and AUD	GA	*✓*	—
[14]	—	—	—	IQD	Yager ranking	—	—
[15]	—	—	—	IQD	Excel macro	—	—
[16]	—	—	*✓*	—	TOPSIS and GA	*✓*	—
[17]	*✓*	—	—	—	ACO	—	—
[18]	—	—	—	—	Tabu search and Lagrangian	—	—
[19]	—	*✓*	—	—	Tabu search	—	—
[20]	—	—	—	IQD	Goal programming and GA	*✓*	—
[21]	*✓*	—	—	IQD	GA and fuzzy simulation	*✓*	—
[22]	*✓*	—	—	—	GA	*✓*	—
[23]	*✓*	—	—	—	SA and GA	*✓*	—
[24]	—	—	*✓*	—	TOPSIS and GA	*✓*	—
[25]	*✓*	—	—	IQD	fuzzy simulation	*✓*	—
[26]	*✓*	—	—	—	Harmony search	*✓*	—
[27]	*✓*	—	—	—	Bee colony and PSO	*✓*	—
[28]	*✓*	—	*✓*	—	Fuzzy programming algorithm	—	—
[29]	—	*✓*	*✓*	—	Fuzzy method	—	—

This research	*✓*	*✓*	*✓*	*✓*	PSO and GA	*✓*	Taguchi

**Table 2:** Different problems and their optimal TMF values obtained by the two algorithms.

Problem	*m*	*N*	*M*_1_	TB	Objective values		MOPSO				MOGA			
					MOPSO	MOGA	*C*_1_	*C*_2_	Pop	Gen	*P*_*C*_	*P*_*m*_	Pop	Gen
1	1	3	2000	30000	18510	18510	2	2.5	30	200	0.6	0.1	40	500
2	2	3	3000	85000	44622	44943	1.5	2.5	20	200	0.5	0.08	30	200
3	3	3	5000	170000	85830	86089	1.5	2	30	500	0.6	0.08	50	300
4	2	4	3500	130000	92758	93423	2	2	30	100	0.7	0.2	50	200
5	3	4	5000	240000	147480	146450	1.5	2.5	40	100	0.6	0.2	50	200
6	4	3	6000	260000	132910	133400	2.5	1.5	20	200	0.6	0.08	40	500
7	5	3	9000	370000	153840	154550	2	2.5	40	200	0.6	0.2	50	200
8	6	3	8800	360000	214620	215510	1.5	2.5	30	500	0.5	0.1	30	200
9	7	3	10500	400000	265020	266020	2	2	30	200	0.7	0.1	40	300
10	8	5	12000	470000	322850	328250	2.5	2.5	30	500	0.6	0.08	50	200
11	8	8	15000	550000	431245	442100	2	2	20	200	0.7	0.2	30	300
12	9	5	15000	530000	438790	449835	1.5	2.5	40	100	0.7	0.2	50	300
13	9	8	18000	600000	495470	513725	2	2	40	500	0.7	0.08	50	500
14	9	9	20000	630000	553276	571250	1.5	2.5	20	100	0.5	0.1	40	500
15	10	5	20000	620000	579030	593167	2.5	2.5	20	200	0.5	0.2	30	200
16	10	8	25000	700000	642870	665890	1.5	2.5	40	200	0.7	0.08	30	200
17	10	10	34000	780000	710035	731280	1.5	1.5	30	200	0.5	0.08	50	500
18	10	15	45000	900000	823210	852400	2	2.5	20	500	0.6	0.1	40	500
19	11	10	40000	850000	827659	859080	1.5	2.5	30	500	0.6	0.1	30	500
20	11	15	45000	870000	867840	897500	2.5	2	40	100	0.7	0.2	50	200
21	12	10	48000	870000	902720	948956	1.5	2.5	30	200	0.5	0.2	50	300
22	12	12	53000	900000	932760	974380	1.5	2	30	100	0.6	0.2	40	200
23	12	15	58000	950000	965470	1023950	2	2.5	20	100	0.5	0.2	30	200
24	13	10	52000	890000	973200	1043569	1.5	1.5	20	500	0.7	0.08	40	300
25	13	13	55000	930000	985439	1089210	1.5	2.5	40	500	0.6	0.1	30	300
26	13	15	62000	980000	1056810	1104325	2	2.5	40	500	0.5	0.1	40	500
27	15	8	57000	900000	1059835	1110360	2	2	20	200	0.6	0.08	50	500
28	15	10	63000	900000	1095430	1176509	2.5	2.5	30	100	0.6	0.2	30	200
29	15	12	68000	950000	1198720	1332900	2.5	1.5	30	500	0.5	0.1	40	200
30	15	15	75000	1000000	1256980	1447905	1.5	2	20	500	0.6	0.08	50	200
31	16	12	70000	940000	1298750	1473400	2	1.5	40	100	0.7	0.1	50	500
32	16	15	80000	1050000	1454328	1772349	1.5	2.5	40	500	0.7	0.2	40	200
33	17	15	87000	1100000	1543890	1809850	2	2	40	200	0.7	0.1	50	500
34	17	17	93000	1140000	1630215	1865780	2.5	2.5	30	500	0.6	0.1	40	300
35	18	10	80000	1000000	1678345	1890437	1.5	1.5	30	100	0.7	0.08	50	300
36	18	15	98000	1150000	1768950	1924670	2	2.5	20	500	0.5	0.08	40	300
37	18	18	103000	1230000	1832450	1987320	1.5	2	30	100	0.7	0.1	40	200
38	20	10	100000	1200000	1876895	1998230	2.5	2.5	20	500	0.6	0.1	50	500
39	20	15	108000	1260000	1904564	2035689	1.5	2	30	500	0.7	0.2	50	500
40	20	20	115000	1330000	1987350	2154670	1.5	1.5	30	100	0.7	0.08	30	500
Mean	—	—	—	—	**897145**	**971541**	—	—	—	—	—	—	—	—
St. Dev	—	—	—	—	**581013**	**652778**	—	—	—	—	—	—	—	—

**Table 3:** The general data for a problem with 5 items and 3 periods.

Product	*D*_*i*,1_	*D*_*i*,2_	*π*_*i*,1_	*π*_*i*,2_	${\hat{\pi }}_{i,1}$	${\hat{\pi }}_{i,2}$	*B*_*i*_	*H*_*i*_	*A*_*i*_	*β*_*i*_	*S*_*i*_
1	1200	800	20	18	9	10	3	5	20	0.5	4
2	1300	900	20	18	9	10	7	5	15	0.5	6
3	1500	1200	11	14	8	12	5	6	25	0.8	7
4	2100	2000	11	14	8	12	8	6	18	0.8	5
5	1800	1600	12	15	9	11	7	7	19	0.6	6

**Table 4:** The parameters of the two algorithms and their levels.

Algorithms	Factors	Levels [1,2, 3]
MOPSO	*C*_1_ (*A*)	[1.5,2, 2.5]
	*C*_2_ (*B*)	[1.5,2, 2.5]
	Pop(*C*)	[20,30,40]
	Gen(*D*)	[100,200,500]

MOGA	*P*_*C*_ (*A*)	[0.5,0.6,0.7]
	*P*_*m*_ (*B*)	[0.08,0.1,0.2]
	Pop(*C*)	[30,40,50]
	Gen(*D*)	[200,300,500]

**Table 5:** The Taguchi *L*_9_ design along with objective values of the algorithms.

Run number	*A*	*B*	*C*	*D*	MOPSO	MOGA
1	1	1	1	1	154040	154980
2	1	2	2	2	154367	154760
3	1	3	3	3	154220	155075
4	2	1	2	3	153944	154875
5	2	2	3	1	153985	155230
6	2	3	1	2	154568	155102
7	3	1	3	2	154215	154780
8	3	2	1	3	154320	154750
9	3	3	2	1	154100	155111

**Table 6:** The optimal levels of the algorithms' parameters for problem 7 of Table 2.

Algorithms	Factors	Optimal levels
MOPSO	*C*_1_	2
	*C*_2_	2.5
	Pop	40
	Gen	200

MOGA	*P*_*C*_	0.6
	*P*_*m*_	0.2
	Pop	50
	Gen	200

**Table 7:** The best result of the MOPSO algorithm.

Product	*Q*_*i*,1_	*Q*_*i*,2_	*X*_*i*,2_	*X*_*i*,3_	*V*_*i*,1_	*V*_*i*,2_	*b*_*i*,1_	*b*_*i*,2_	TMF
1	1215	159	15	0	405	53	0	626	153840
2	1162	252	0	0	166	36	138	786	
3	1555	190	55	0	311	38	0	955	
4	1360	864	0	0	170	108	740	1876	
5	1435	420	0	0	205	60	365	1545	

**Table 8:** The best result of the MOGA algorithm.

Product	*Q*_*i*,1_	*Q*_*i*,2_	*X*_*i*,2_	*X*_*i*,3_	*V*_*i*,1_	*V*_*i*,2_	*b*_*i*,1_	*b*_*i*,2_	TMF
1	1221	168	21	0	407	56	0	611	154550
2	959	392	0	0	137	56	341	849	
3	1220	390	0	0	244	78	280	1090	
4	1168	960	0	0	146	120	932	1972	
5	168	2254	0	0	24	322	1632	978	

**Table 9:** The ANOVA analysis of the performances.

Source	DF	SS	MS	*F*	*P* value
Factor	1	1.11*E* + 11	1.11*E* + 11	0.28	0.6
Error	78	3.11*E* + 13	3.99*E* + 11	—	—

Total	79	3.12*E* + 13	—	—	—
